# Supplementary material for: Pancreatic cancer is associated with medication changes prior to clinical diagnosis
Source: Nat Commun. 2023 Apr 28;14:2437. doi: 10.1038/s41467-023-38088-2 (PMC10147931; doi:10.1038/s41467-023-38088-2)
Supplement: Supplementary file 1 — Supplementary Information [file 41467_2023_38088_MOESM1_ESM.pdf]

SUPPLEMENTARY INFORMATION

Pancreatic cancer is associated with medication changes prior to clinical diagnosis

Yin Zhang, Qiao-Li Wang, Chen Yuan, Alice A. Lee, Ana Babic, Kimmie Ng, Kimberly Perez, Jonathan A. Nowak, Jesper Lagergren, Meir J. Stampfer, Edward L. Giovannucci, Chris Sander, Michael H. Rosenthal, Peter Kraft, Brian M. Wolpin

Supplementary Table 1. Summary of study populations by medication use category

Supplementary Table 2. Characteristics of participants according to antidiabetic, anticoagulant, and antihypertensive medication use in the pooled NHS and HPFS cohorts

Supplementary Table 3. Characteristics of participants according to antacid, NSAIDs, and antidepressant medication use in the pooled NHS and HPFS cohorts

Supplementary Table 4. Recent change in antidiabetic, anticoagulant, and antihypertensive medication use and 2-year risk of pancreatic cancer diagnosis in the pooled NHS and HPFS cohorts

Supplementary Table 5. Recent change in antacid, NSAIDs, and antidepressant medication use and 2-year risk of pancreatic cancer diagnosis in the pooled NHS and HPFS cohorts

Supplementary Table 6. Sensitivity analysis of recent change in antidiabetic, anticoagulant, and antihypertensive medication use and 2-year risk of pancreatic cancer diagnosis in the pooled NHS and HPFS cohorts with four-category medication change exposure variable

Supplementary Table 7. Sensitivity analysis of recent change in antacid, NSAIDs, and antidepressant medication use and 2-year risk of pancreatic cancer diagnosis in the pooled NHS and HPFS cohorts with four-category medication change exposure variable

Supplementary Table 8. Recent change in antidiabetic, anticoagulant, and antihypertensive medication use and 2-year risk of pancreatic cancer diagnosis in the NHS cohort

Supplementary Table 9. Recent change in antacid, NSAIDs, and antidepressant medication use and 2-year risk of pancreatic cancer diagnosis in the NHS cohort

Supplementary Table 10. Recent change in anticoagulant and antihypertensive medication use and 2-year risk of pancreatic cancer diagnosis in the HPFS cohort

Supplementary Table 11. Recent change in antacid, NSAIDs, and antidepressant medication use and 2-year risk of pancreatic cancer diagnosis in the HPFS cohort

Supplementary Table 12. Combined associations of recent changes in medication use with 2-year risk of pancreatic cancer diagnosis in the NHS cohort

Supplementary Table 13. Recent change in medication use and 2-year risk of other digestive system cancer diagnosis in the pooled NHS and HPFS cohorts

Supplementary Table 1. Summary of study populations by medication use category

Medication under study	Timelines of exposure assessments	Timelines of analyses [a]	Eligible participants	Person-years of follow-up	Incident pancreatic cancer cases
Antidiabetic medications	NHS: 1988-2010<br>HPFS: Not included [b]	NHS: 1990-2012<br>HPFS: Not included [b]	101,294	2,015,712	588
Anticoagulant medications	NHS: 1994-2010<br>HPFS: 1996-2010	NHS: 1996-2012<br>HPFS: 1998-2012	128,718	1,791,733	688
Antihypertensive medications	NHS: 1988-2010<br>HPFS: 1986-2010	NHS: 1990-2012<br>HPFS: 1988-2012	148,973	2,994,057	991
Antacids	NHS: 2000-2010<br>HPFS: 2004-2010	NHS: 2002-2012<br>HPFS: 2006-2012	83,611	648,333	269
NSAIDs	NHS: 1990-2010<br>HPFS: 1986-2010	NHS: 1992-2012<br>HPFS: 1988-2012	145,883	2,611,575	903
Antidepressants	NHS: 1996-2010<br>HPFS: 1990-2010	NHS: 1998-2012<br>HPFS: 1992-2012	131,787	1,880,573	755
Combined antidiabetic, anticoagulant, and antihypertensive medications	NHS: 1994-2010<br>HPFS: Not included [b]	NHS: 1996-2012<br>HPFS: Not included [b]	84,623	1,128,153	376

Abbreviations: NHS, Nurses' Health Study; HPFS, Health Professionals Follow-up Study; NSAIDs, non-steroidal anti-inflammatory drugs.

[a] Medication change in the prediagnosis time window was measured by comparing questionnaires returned at a median of 1 year (current status of medication use) and 3 years (previous status of medication use) before diagnosis. Therefore, 2-year intervals were present between baseline of analyses and the time when information on use of medications was first assessed.

[b] Information on antidiabetic medication use in HPFS was assessed in 2008 and afterwards. Given the short interval with available data, antidiabetic medication use in HPFS was not included in the analyses of change in antidiabetic medication use and the combined associations of starting antidiabetic, starting anticoagulant, and stopping antihypertensive medications.

Supplementary Table 2. Characteristics of participants according to antidiabetic, anticoagulant, and antihypertensive medication use in the NHS and HPFS cohorts

Characteristic [a,b,c,d]	OHG (NHS)		Insulin (NHS)		Anticoagulant (Pooled NHS and HPFS)		Antihypertensive medication (Pooled NHS and HPFS)	
	No	Yes	No	Yes	No	Yes	No	Yes
Age, years, mean (SD)	65.7 (9.3)	69.8 (7.7)	65.8 (9.3)	68.8 (8.2)	68.7 (8.5)	74.9 (7.5)	64.3 (10.0)	68.8 (8.6)
Caucasian, %	97.0	95.8	97.0	95.8	95.4	96.0	94.6	95.4
BMI, kg/m^2, mean (SD) [e]	25.3 (4.4)	30.7 (5.4)	25.4 (4.4)	29.6 (6.0)	25.7 (4.2)	27.3 (5.2)	25.2 (3.9)	26.6 (4.6)
Pack-years of smoking, mean (SD) [f]	13.4 (20.0)	13.9 (20.5)	13.4 (20.0)	14.1 (20.7)	12.7 (19.4)	13.8 (19.7)	12.7 (19.4)	13.3 (19.5)
History of diabetes mellitus, %	6.6	97.8	8.5	99.3	11.0	18.3	7.4	13.7
Physical activity, MET-hours/week, mean (SD) [g]	16.9 (16.9)	13.0 (12.9)	16.8 (16.9)	13.1 (14.4)	20.9 (19.6)	19.3 (18.0)	21.5 (21.1)	18.8 (17.8)
Alcohol, g/day, mean (SD)	5.6 (8.5)	2.8 (5.9)	5.5 (8.5)	2.6 (6.0)	6.8 (10.0)	7.3 (10.8)	7.2 (10.7)	7.3 (11.0)
Multivitamin use, %	49.8	56.2	50.0	54.6	56.4	60.0	44.5	58.7

Abbreviations: NHS, Nurses' Health Study; HPFS, Health Professionals Follow-up Study; SD, standard deviation; BMI, body mass index; MET, metabolic equivalent task; OHG, oral hypoglycemic agent.

[a] Follow-up time: antidiabetic medications (NHS: 1990-2012; information on antidiabetic medication use in HPFS was assessed relatively late (in 2008 and afterwards) and was therefore not included in the analyses), anticoagulant (NHS: 1996-2012; HPFS: 1998-2012), and antihypertensive medications (NHS: 1990-2012; HPFS: 1988-2012).
[b] All variables are age-standardized except age.
[c] Percentages are of non-missing values.
[d] Percentages may not sum to 100% after rounding.
[e] Calculated as weight in kilograms divided by height in meters squared.
[f] Cumulative among ever smokers.
[g] Weekly energy expenditure in MET-hours/week from recreational and leisure-time physical activity.

Supplementary Table 3. Characteristics of participants according to antacid, NSAIDs, and antidepressant medication use in the NHS and HPFS cohorts

Characteristic [a,b,c,d]	H2 blocker (Pooled NHS and HPFS)		Proton pump inhibitor (Pooled NHS and HPFS)		NSAIDs (Pooled NHS and HPFS)		Antidepressant (Pooled NHS and HPFS)	
	No	Yes	No	Yes	No	Yes	No	Yes
Age, years, mean (SD)	71.6 (7.5)	71.6 (7.3)	71.6 (7.5)	71.9 (7.3)	65.1 (9.9)	67.1 (9.3)	68.6 (9.1)	68.4 (8.2)
Caucasian, %	96.6	97.5	96.6	97.4	94.5	95.7	94.3	97.3
BMI, kg/m^2, mean (SD) [e]	25.5 (4.2)	26.4 (4.5)	25.5 (4.2)	26.3 (4.4)	25.1 (3.9)	25.9 (4.3)	25.7 (4.1)	26.3 (4.6)
Pack-years of smoking, mean (SD) [f]	12.0 (18.8)	13.6 (19.7)	12.0 (18.9)	12.4 (18.7)	12.2 (19.0)	13.4 (19.8)	12.3 (19.0)	15.1 (21.1)
History of diabetes mellitus, %	11.5	14.5	11.2	14.3	8.0	10.6	10.5	14.9
Physical activity, MET-hours/week, mean (SD) [g]	21.2 (18.6)	18.1 (15.9)	21.5 (18.9)	18.5 (16.3)	21.3 (20.2)	20.8 (19.9)	23.0 (20.8)	17.5 (16.5)
Alcohol, g/day, mean (SD)	6.8 (9.6)	6.2 (9.1)	6.9 (9.6)	6.2 (9.2)	6.8 (10.3)	7.5 (10.9)	7.7 (11.0)	6.6 (9.7)
Multivitamin use, %	70.2	72.3	69.9	72.7	53.0	52.1	54.5	68.0

Abbreviations: NHS, Nurses' Health Study; HPFS, Health Professionals Follow-up Study; SD, standard deviation; BMI, body mass index; MET, metabolic equivalent task; NSAIDs, non-steroidal anti-inflammatory drugs.

[a] Follow-up time: antacids (NHS: 2002-2012; HPFS: 2006-2012), NSAIDs (NHS: 1992-2012; HPFS: 1988-2012), and antidepressants (NHS: 1998-2012; HPFS: 1992-2012).
[b] All variables are age-standardized except age.
[c] Percentages are of non-missing values.
[d] Percentages may not sum to 100% after rounding.
[e] Calculated as weight in kilograms divided by height in meters squared.
[f] Cumulative among ever smokers.
[g] Weekly energy expenditure in MET-hours/week from recreational and leisure time physical activity.

Supplementary Table 4. Recent change in antidiabetic, anticoagulant, and antihypertensive medication use and 2-year risk of pancreatic cancer diagnosis in the pooled NHS and HPFS cohorts

	Recent change in medication use [a,b]		
	No change	Start	Stop
Antidiabetic medications			
No. of cases	538	33	17
Person-years	1,966,617	33,435	15,660
Incidence rate (per 100,000 person-years)	27 (25-30)	99 (70-139)	109 (67-175)
Unadjusted	1 [Ref]	3.61 (2.54-5.13)	3.97 (2.45-6.43)
Age-adjusted [c]	1	2.99 (2.10-4.27)	2.76 (1.70-4.50)
Age-adjusted [c] (+ BMI + history of diabetes)	1	1.93 (1.28-2.91)	1.77 (1.04-3.01)
MV-adjusted [d]	1	1.99 (1.30-3.03)	1.55 (0.86-2.81)
MV-adjusted [e] (replace BMI with weight loss)	1	2.13 (1.40-3.25)	1.38 (0.76-2.50)
Oral hypoglycemic medications			
No. of cases	549	22	17
Person-years	1,974,953	26,767	13,992
Incidence rate (per 100,000 person-years)	28 (26-30)	82 (54-125)	121 (76-195)
Unadjusted	1	2.96 (1.93-4.53)	4.37 (2.70-7.08)
Age-adjusted [c]	1	2.40 (1.56-3.69)	3.11 (1.91-5.06)
Age-adjusted [c] (+ BMI + history of diabetes)	1	1.47 (0.92-2.34)	1.87 (1.11-3.16)
MV-adjusted [d]	1	1.39 (0.86-2.27)	2.04 (1.09-3.82)
MV-adjusted [e] (replace BMI with weight loss)	1	1.49 (0.92-2.44)	1.87 (1.00-3.50)
Insulin			
No. of cases	570	13	5
Person-years	2,002,769	8,653	4,290
Incidence rate (per 100,000 person-years)	28 (26-31)	150 (87-259)	117 (49-280)
Unadjusted	1	5.28 (3.05-9.15)	4.10 (1.70-9.88)
Age-adjusted [c]	1	4.69 (2.69-8.18)	2.83 (1.17-6.87)
Age-adjusted [c] (+ BMI + history of diabetes)	1	2.74 (1.53-4.91)	1.67 (0.68-4.11)
MV-adjusted [d]	1	2.84 (1.58-5.12)	1.18 (0.43-3.24)
MV-adjusted [e] (replace BMI with weight loss)	1	3.10 (1.72-5.59)	0.99 (0.36-2.73)
Anticoagulant medications			
No. of cases	658	20	10
Person-years	1,754,044	25,461	12,227
Incidence rate (per 100,000 person-years)	38 (35-40)	79 (51-122)	82 (44-152)
Unadjusted	1	2.09 (1.34-3.27)	2.18 (1.17-4.07)
Age-adjusted [c]	1	1.60 (1.02-2.52)	1.52 (0.81-2.87)
MV-adjusted [d]	1	1.50 (0.95-2.35)	1.18 (0.57-2.46)
MV-adjusted [e] (replace BMI with weight loss)	1	1.50 (0.96-2.36)	1.08 (0.52-2.26)
Antihypertensive medications			
No. of cases	789	86	116
Person-years	2,627,927	224,273	141,857
Incidence rate (per 100,000 person-years)	30 (28-32)	38 (31-47)	82 (68-98)
Unadjusted	1	1.28 (1.02-1.60)	2.72 (2.24-3.31)
Age-adjusted [c]	1	1.04 (0.83-1.31)	1.95 (1.60-2.38)
MV-adjusted [d]	1	1.03 (0.81-1.30)	1.77 (1.42-2.20)
MV-adjusted [e] (replace BMI with weight loss)	1	1.08 (0.86-1.37)	1.63 (1.30-2.03)

Abbreviations: NHS, Nurses' Health Study; HPFS, Health Professionals Follow-up Study; MV, multivariate; BMI, body mass index; MET, metabolic equivalent task.

[a] Follow-up time: antidiabetic medications (NHS: 1990-2012; information on antidiabetic medication use in HPFS was assessed relatively late (in 2008 and afterwards) and was therefore not included in the analyses), anticoagulant (NHS: 1996-2012; HPFS: 1998-2012), and antihypertensive medications (NHS: 1990-2012; HPFS: 1988-2012).

[b] Medication change was measured by comparing questionnaires returned at a median of 1 year (current status of use) and 3 years (previous status of use) before pancreatic cancer diagnosis.

[c] Stratified by age (in months), sex/cohort (women, men; in the pooled analyses only), and calendar year of the survey cycle (each 2-year interval).

[d] Stratified by age (in months), sex/cohort (women, men; in the pooled analyses only), and calendar year of the survey cycle (each 2-year interval); adjusted for race/ethnicity (white, black, other, unknown), BMI (continuous, kg/m^2), physical activity (continuous, MET-hours/week), smoking (continuous, pack-years), alcohol intake (continuous, grams/day), history of diabetes (yes, no), multivitamin use (yes, no), and previous status of use (yes, no).

[e] Replace BMI (continuous, kg/m^2) with recent weight loss (no, ≤5%, 5%< to ≤ 10%, and >10% weight loss).

Supplementary Table 5. Recent change in antacid, NSAIDs, and antidepressant medication use and 2-year risk of pancreatic cancer diagnosis in the pooled NHS and HPFS cohorts

	Recent change in medication use [a,b]		
	No change	Start	Stop
Antacids			
No. of cases	229	21	19
Person-years	539,960	62,898	45,475
Incidence rate (per 100,000 person-years)	42 (37-48)	33 (22-51)	42 (27-66)
Unadjusted	1 [Ref]	0.79 (0.50-1.23)	0.99 (0.62-1.57)
Age-adjusted [c]	1	0.81 (0.52-1.28)	1.01 (0.63-1.62)
MV-adjusted [d]	1	0.76 (0.48-1.21)	1.06 (0.58-1.93)
MV-adjusted [e] (replace BMI with weight loss)	1	0.76 (0.48-1.20)	1.04 (0.57-1.90)
NSAIDs			
No. of cases	800	49	54
Person-years	2,333,006	148,689	129,881
Incidence rate (per 100,000 person-years)	34 (32-37)	33 (25-44)	42 (32-54)
Unadjusted	1	0.96 (0.72-1.28)	1.21 (0.92-1.60)
Age-adjusted [c]	1	0.96 (0.71-1.29)	1.17 (0.88-1.55)
MV-adjusted [d]	1	1.04 (0.76-1.42)	1.14 (0.85-1.52)
MV-adjusted [e] (replace BMI with weight loss)	1	1.01 (0.74-1.38)	1.15 (0.86-1.54)
Antidepressants			
No. of cases	707	20	28
Person-years	1,790,806	51,326	38,441
Incidence rate (per 100,000 person-years)	39 (37-42)	39 (25-60)	73 (50-105)
Unadjusted	1	0.99 (0.63-1.54)	1.84 (1.26-2.69)
Age-adjusted [c]	1	1.05 (0.67-1.65)	1.88 (1.28-2.75)
MV-adjusted [d]	1	0.99 (0.63-1.55)	1.39 (0.85-2.27)
MV-adjusted [e] (replace BMI with weight loss)	1	0.99 (0.63-1.55)	1.30 (0.79-2.13)

Abbreviations: NHS, Nurses' Health Study; HPFS, Health Professionals Follow-up Study; MV, multivariate; BMI, body mass index; MET, metabolic equivalent task; NSAIDs, non-steroidal anti-inflammatory drugs.

[a] Follow-up time: antacids (NHS: 2002-2012; HPFS: 2006-2012), NSAIDs (NHS: 1992-2012; HPFS: 1988-2012), and antidepressants (NHS: 1998-2012; HPFS: 1992-2012).

[b] Medication change was measured by comparing questionnaires returned at a median of 1 year (current status of use) and 3 years (previous status of use) before pancreatic cancer diagnosis.

[c] Stratified by age (in months), sex/cohort (women, men; in the pooled analyses only), and calendar year of the survey cycle (each 2-year interval).

[d] Stratified by age (in months), sex/cohort (women, men; in the pooled analyses only), and calendar year of the survey cycle (each 2-year interval); adjusted for race/ethnicity (white, black, other, unknown), BMI (continuous, kg/m^2), physical activity (continuous, MET-hours/week), smoking (continuous, pack-years), alcohol intake (continuous, grams/day), history of diabetes (yes, no), multivitamin use (yes, no), and previous status of use (yes, no).

[e] Replace BMI (continuous, kg/m^2) with recent weight loss (no, ≤5%, 5%< to ≤ 10%, and >10% weight loss).

Supplementary Table 6. Sensitivity analysis of recent change in antidiabetic, anticoagulant, and antihypertensive medication use and 2-year risk of pancreatic cancer diagnosis in the pooled NHS and HPFS cohorts with four-category medication change exposure variable

	Recent change in medication use [a,b]			
	Not use and stable	Use and stable	Start	Stop
Antidiabetic medications				
No. of cases	507	31	33	17
Person-years	1,910,113	56,504	33,435	15,660
Incidence rate (per 100,000 person-years)	27 (24-29)	55 (39-78)	99 (70-139)	109 (67-175)
Unadjusted	1 [Ref]	2.07 (1.44-2.97)	3.72 (2.61-5.29)	4.09 (2.52-6.63)
Age-adjusted [c]	1	1.69 (1.17-2.43)	3.07 (2.15-4.38)	2.84 (1.74-4.63)
Age-adjusted [c] (+ BMI + history of diabetes)	1	1.08 (0.68-1.71)	1.99 (1.27-3.13)	1.83 (1.04-3.21)
MV-adjusted [d]	1	0.78 (0.35-1.74)	1.87 (1.17-2.98)	1.24 (0.49-3.15)
MV-adjusted [e] (replace BMI with weight loss)	1	0.84 (0.38-1.89)	2.05 (1.28-3.26)	1.19 (0.47-3.03)
Oral hypoglycemic medications				
No. of cases	525	24	22	17
Person-years	1,930,135	44,818	26,767	13,992
Incidence rate (per 100,000 person-years)	27 (25-30)	54 (36-80)	82 (54-125)	121 (76-195)
Unadjusted	1	1.97 (1.31-2.96)	3.02 (1.97-4.63)	4.47 (2.76-7.24)
Age-adjusted [c]	1	1.56 (1.03-2.35)	2.44 (1.59-3.76)	3.17 (1.94-5.16)
Age-adjusted [c] (+ BMI + history of diabetes)	1	0.88 (0.55-1.41)	1.41 (0.87-2.30)	1.80 (1.05-3.09)
MV-adjusted [d]	1	0.86 (0.54-1.39)	1.39 (0.86-2.27)	1.76 (1.03-3.03)
MV-adjusted [e] (replace BMI with weight loss)	1	0.97 (0.60-1.56)	1.49 (0.92-2.44)	1.81 (1.05-3.10)
Insulin				
No. of cases	554	16	13	5
Person-years	1,981,445	21,324	8,653	4,290
Incidence rate (per 100,000 person-years)	28 (26-30)	75 (46-122)	150 (87-259)	117 (49-280)
Unadjusted	1	2.68 (1.63-4.41)	5.37 (3.10-9.31)	4.17 (1.73-10.1)
Age-adjusted [c]	1	2.39 (1.45-3.95)	4.77 (2.74-8.32)	2.89 (1.19-7.00)
Age-adjusted [c] (+ BMI + history of diabetes)	1	1.45 (0.85-2.47)	2.88 (1.60-5.18)	1.75 (0.71-4.33)
MV-adjusted [d]	1	1.43 (0.84-2.45)	2.84 (1.58-5.12)	1.69 (0.68-4.19)
MV-adjusted [e] (replace BMI with weight loss)	1	1.54 (0.90-2.64)	3.10 (1.72-5.59)	1.53 (0.62-3.79)
Anticoagulant medications				
No. of cases	631	27	20	10
Person-years	1,717,186	36,859	25,461	12,227
Incidence rate (per 100,000 person-years)	37 (34-40)	73 (50-107)	79 (51-122)	82 (44-152)
Unadjusted	1	1.99 (1.36-2.93)	2.14 (1.37-3.34)	2.23 (1.19-4.16)
Age-adjusted [c]	1	1.26 (0.85-1.87)	1.62 (1.03-2.55)	1.54 (0.82-2.91)
MV-adjusted [d]	1	1.16 (0.78-1.72)	1.50 (0.95-2.35)	1.37 (0.73-2.59)
MV-adjusted [e] (replace BMI with weight loss)	1	1.21 (0.82-1.79)	1.50 (0.96-2.36)	1.31 (0.69-2.47)
Antihypertensive medications				
No. of cases	503	286	86	116
Person-years	1,933,444	694,483	224,273	141,857
Incidence rate (per 100,000 person-years)	26 (24-28)	41 (37-46)	38 (31-47)	82 (68-98)
Unadjusted	1	1.58 (1.37-1.83)	1.47 (1.17-1.85)	3.14 (2.57-3.85)
Age-adjusted [c]	1	1.16 (1.00-1.35)	1.10 (0.87-1.39)	2.06 (1.68-2.54)
MV-adjusted [d]	1	1.06 (0.91-1.24)	1.03 (0.81-1.30)	1.88 (1.52-2.32)
MV-adjusted [e] (replace BMI with weight loss)	1	1.14 (0.98-1.34)	1.08 (0.86-1.37)	1.87 (1.51-2.30)

Abbreviations: NHS, Nurses' Health Study; HPFS, Health Professionals Follow-up Study; MV, multivariate; BMI, body mass index; MET, metabolic equivalent task.

[a] Follow-up time: antidiabetic medications (NHS: 1990-2012; information on antidiabetic medication use in HPFS was assessed relatively late (in 2008 and afterwards) and was therefore not included in the analyses), anticoagulant (NHS: 1996-2012; HPFS: 1998-2012), and antihypertensive medications (NHS: 1990-2012; HPFS: 1988-2012).

[b] Medication change was measured by comparing questionnaires returned at a median of 1 year (current status of use) and 3 years (previous status of use) before pancreatic cancer diagnosis. Stable refers to medication use or non-use that remained unchanged in the two-year time window, which includes two possible scenarios: stable use and stable non-use over the two years.

[c] Stratified by age (in months), sex/cohort (women, men; in the pooled analyses only), and calendar year of the survey cycle (each 2-year interval).

[d] Stratified by age (in months), sex/cohort (women, men; in the pooled analyses only), and calendar year of the survey cycle (each 2-year interval); adjusted for race/ethnicity (white, black, other, unknown), BMI (continuous, kg/m^2), physical activity (continuous, MET-hours/week), smoking (continuous, pack-years), alcohol intake (continuous, grams/day), history of diabetes (yes, no), multivitamin use (yes, no), and previous status of use (yes, no).

[e] Replace BMI (continuous, kg/m^2) with recent weight loss (no, ≤5%, 5%< to ≤ 10%, and >10% weight loss).

Supplementary Table 7. Sensitivity analysis of recent change in antacid, NSAIDs, and antidepressant medication use and 2-year risk of pancreatic cancer diagnosis in the pooled NHS and HPFS cohorts with four-category medication change exposure variable

	Recent change in medication use [a,b]			
	Not use and stable	Use and stable	Start	Stop
Antacids				
No. of cases	202	27	21	19
Person-years	470,356	69,604	62,898	45,475
Incidence rate (per 100,000 person-years)	43 (37-49)	39 (27-57)	33 (22-51)	42 (27-66)
Unadjusted	1 [Ref]	0.90 (0.60-1.35)	0.78 (0.50-1.22)	0.97 (0.61-1.56)
Age-adjusted [c]	1	0.89 (0.60-1.34)	0.80 (0.51-1.26)	0.99 (0.62-1.60)
MV-adjusted [d]	1	0.96 (0.37-2.48)	0.76 (0.47-1.23)	1.03 (0.37-2.88)
MV-adjusted [e] (replace BMI with weight loss)	1	0.96 (0.37-2.48)	0.75 (0.46-1.23)	1.01 (0.36-2.84)
NSAIDs				
No. of cases	283	517	49	54
Person-years	981,890	1,351,116	148,689	129,881
Incidence rate (per 100,000 person-years)	29 (26-32)	38 (35-42)	33 (25-44)	42 (32-54)
Unadjusted	1	1.33 (1.15-1.53)	1.14 (0.84-1.55)	1.44 (1.08-1.93)
Age-adjusted [c]	1	1.17 (1.01-1.35)	1.06 (0.77-1.44)	1.29 (0.95-1.74)
MV-adjusted [d]	1	1.10 (0.95-1.28)	1.04 (0.76-1.42)	1.25 (0.92-1.69)
MV-adjusted [e] (replace BMI with weight loss)	1	1.08 (0.93-1.25)	1.01 (0.74-1.38)	1.24 (0.92-1.68)
Antidepressants				
No. of cases	669	38	20	28
Person-years	1,711,778	79,028	51,326	38,441
Incidence rate (per 100,000 person-years)	39 (36-42)	48 (35-66)	39 (25-60)	73 (50-105)
Unadjusted	1	1.23 (0.89-1.71)	1.00 (0.64-1.56)	1.86 (1.28-2.72)
Age-adjusted [c]	1	1.39 (1.00-1.94)	1.07 (0.68-1.68)	1.91 (1.31-2.81)
MV-adjusted [d]	1	1.28 (0.91-1.78)	0.99 (0.63-1.55)	1.77 (1.20-2.59)
MV-adjusted [e] (replace BMI with weight loss)	1	1.31 (0.94-1.84)	0.99 (0.63-1.55)	1.71 (1.16-2.51)

Abbreviations: NHS, Nurses' Health Study; HPFS, Health Professionals Follow-up Study; MV, multivariate; BMI, body mass index; MET, metabolic equivalent task; NSAIDs, non-steroidal anti-inflammatory drugs.

[a] Follow-up time: antacids (NHS: 2002-2012; HPFS: 2006-2012), NSAIDs (NHS: 1992-2012; HPFS: 1988-2012), and antidepressants (NHS: 1998-2012; HPFS: 1992-2012).

[b] Medication change was measured by comparing questionnaires returned at a median of 1 year (current status of use) and 3 years (previous status of use) before pancreatic cancer diagnosis. Stable refers to medication use or non-use that remained unchanged in the two-year time window, which includes two possible scenarios: stable use and stable non-use over the two years.

[c] Stratified by age (in months), sex/cohort (women, men; in the pooled analyses only), and calendar year of the survey cycle (each 2-year interval).

[d] Stratified by age (in months), sex/cohort (women, men; in the pooled analyses only), and calendar year of the survey cycle (each 2-year interval); adjusted for race/ethnicity (white, black, other, unknown), BMI (continuous, kg/m^2), physical activity (continuous, MET-hours/week), smoking (continuous, pack-years), alcohol intake (continuous, grams/day), history of diabetes (yes, no), multivitamin use (yes, no), and previous status of use (yes, no).

[e] Replace BMI (continuous, kg/m^2) with recent weight loss (no, ≤5%, 5%< to ≤ 10%, and >10% weight loss).

Supplementary Table 8. Recent change in antidiabetic, anticoagulant, and antihypertensive medication use and 2-year risk of pancreatic cancer diagnosis in the NHS cohort

	Recent change in medication use [a,b]		
	No change	Start	Stop
Antidiabetic medications			
No. of cases	538	33	17
Person-years	1,966,617	33,435	15,660
Incidence rate (per 100,000 person-years)	27 (25-30)	99 (70-139)	109 (67-175)
Unadjusted	1 [Ref]	3.61 (2.54-5.13)	3.97 (2.45-6.43)
Age-adjusted [c]	1	2.99 (2.10-4.27)	2.76 (1.70-4.50)
MV-adjusted [d]	1	1.99 (1.30-3.04)	1.55 (0.86-2.81)
MV-adjusted [e] (replace BMI with weight loss)	1	2.13 (1.40-3.26)	1.38 (0.76-2.51)
Oral hypoglycemic medications			
No. of cases	549	22	17
Person-years	1,974,953	26,767	13,992
Incidence rate (per 100,000 person-years)	28 (26-30)	82 (54-125)	121 (76-195)
Unadjusted	1	2.96 (1.93-4.53)	4.37 (2.70-7.08)
Age-adjusted [c]	1	2.40 (1.56-3.69)	3.11 (1.91-5.06)
MV-adjusted [d]	1	1.40 (0.86-2.27)	2.04 (1.09-3.82)
MV-adjusted [e] (replace BMI with weight loss)	1	1.50 (0.92-2.44)	1.87 (1.00-3.50)
Insulin			
No. of cases	570	13	5
Person-years	2,002,769	8,653	4,290
Incidence rate (per 100,000 person-years)	28 (26-31)	150 (87-259)	117 (49-280)
Unadjusted	1	5.28 (3.05-9.15)	4.10 (1.70-9.88)
Age-adjusted [c]	1	4.69 (2.69-8.18)	2.83 (1.17-6.87)
MV-adjusted [d]	1	2.84 (1.58-5.12)	1.18 (0.43-3.24)
MV-adjusted [e] (replace BMI with weight loss)	1	3.10 (1.72-5.59)	0.99 (0.36-2.73)
Anticoagulant medications			
No. of cases	444	15	6
Person-years	1,306,962	17,448	8,517
Incidence rate (per 100,000 person-years)	34 (31-37)	86 (52-143)	70 (32-157)
Unadjusted	1	2.53 (1.51-4.23)	2.07 (0.93-4.64)
Age-adjusted [c]	1	1.97 (1.17-3.31)	1.55 (0.69-3.49)
MV-adjusted [d]	1	1.83 (1.09-3.08)	1.15 (0.44-2.99)
MV-adjusted [e] (replace BMI with weight loss)	1	1.85 (1.10-3.12)	1.04 (0.40-2.69)
Antihypertensive medications			
No. of cases	454	58	76
Person-years	1,767,534	154,675	93,504
Incidence rate (per 100,000 person-years)	26 (23-28)	37 (29-49)	81 (65-102)
Unadjusted	1	1.46 (1.11-1.92)	3.16 (2.48-4.03)
Age-adjusted [c]	1	1.21 (0.92-1.59)	2.30 (1.79-2.95)
MV-adjusted [d]	1	1.21 (0.90-1.61)	2.05 (1.57-2.69)
MV-adjusted [e] (replace BMI with weight loss)	1	1.32 (0.98-1.76)	1.85 (1.40-2.43)

Abbreviations: NHS, Nurses' Health Study; MV, multivariate; BMI, body mass index; MET, metabolic equivalent task.

[a] Follow-up time: antidiabetic medications (NHS: 1990-2012), anticoagulant (NHS: 1996-2012), and antihypertensive medications (NHS: 1990-2012).

[b] Medication change was measured by comparing questionnaires returned at a median of 1 year (current status of use) and 3 years (previous status of use) before pancreatic cancer diagnosis.

[c] Stratified by age (in months) and calendar year of the survey cycle (each 2-year interval).

[d] Stratified by age (in months) and calendar year of the survey cycle (each 2-year interval); adjusted for race/ethnicity (white, black, other, unknown), BMI (continuous, kg/m^2), physical activity (continuous, MET-hours/week), smoking (continuous, pack-years), alcohol intake (continuous, grams/day), history of diabetes (yes, no), multivitamin use (yes, no), and previous status of use (yes, no).

[e] Replace BMI (continuous, kg/m^2) with recent weight loss (no, ≤5%, 5%< to ≤ 10%, and >10% weight loss).

Supplementary Table 9. Recent change in antacid, NSAIDs, and antidepressant medication use and 2-year risk of pancreatic cancer diagnosis in the NHS cohort

	Recent change in medication use [a,b]		
	No change	Start	Stop
Antacids			
No. of cases	187	16	14
Person-years	445,780	55,671	38,415
Incidence rate (per 100,000 person-years)	42 (36-48)	29 (18-47)	36 (22-62)
Unadjusted	1 [Ref]	0.69 (0.41-1.14)	0.87 (0.50-1.50)
Age-adjusted [c]	1	0.70 (0.42-1.18)	0.91 (0.53-1.58)
MV-adjusted [d]	1	0.67 (0.40-1.14)	0.92 (0.47-1.81)
MV-adjusted [e] (replace BMI with weight loss)	1	0.67 (0.40-1.14)	0.90 (0.46-1.76)
NSAIDs			
No. of cases	532	15	15
Person-years	1,661,939	57,172	63,333
Incidence rate (per 100,000 person-years)	32 (29-35)	26 (16-44)	24 (14-39)
Unadjusted	1	0.82 (0.49-1.37)	0.74 (0.44-1.24)
Age-adjusted [c]	1	0.92 (0.55-1.54)	0.89 (0.53-1.49)
MV-adjusted [d]	1	1.03 (0.60-1.74)	0.84 (0.50-1.41)
MV-adjusted [e] (replace BMI with weight loss)	1	0.94 (0.55-1.60)	0.86 (0.51-1.44)
Antidepressants			
No. of cases	393	17	19
Person-years	1,050,746	38,488	29,469
Incidence rate (per 100,000 person-years)	37 (34-41)	44 (27-71)	64 (41-101)
Unadjusted	1	1.18 (0.73-1.92)	1.72 (1.09-2.73)
Age-adjusted [c]	1	1.27 (0.78-2.07)	1.76 (1.11-2.81)
MV-adjusted [d]	1	1.18 (0.73-1.93)	1.54 (0.84-2.80)
MV-adjusted [e] (replace BMI with weight loss)	1	1.20 (0.73-1.96)	1.42 (0.78-2.60)

Abbreviations: NHS, Nurses' Health Study; MV, multivariate; BMI, body mass index; MET, metabolic equivalent task; NSAIDs, non-steroidal anti-inflammatory drugs.

[a] Follow-up time: antacids (NHS: 2002-2012), NSAIDs (NHS: 1992-2012), and antidepressants (NHS: 1998-2012).

[b] Medication change was measured by comparing questionnaires returned at a median of 1 year (current status of use) and 3 years (previous status of use) before pancreatic cancer diagnosis.

[c] Stratified by age (in months) and calendar year of the survey cycle (each 2-year interval).

[d] Stratified by age (in months) and calendar year of the survey cycle (each 2-year interval); adjusted for race/ethnicity (white, black, other, unknown), BMI (continuous, kg/m^2), physical activity (continuous, MET-hours/week), smoking (continuous, pack-years), alcohol intake (continuous, grams/day), history of diabetes (yes, no), multivitamin use (yes, no), and previous status of use (yes, no).

[e] Replace BMI (continuous, kg/m^2) with recent weight loss (no, ≤5%, 5%< to ≤ 10%, and >10% weight loss).

Supplementary Table 10. Recent change in anticoagulant and antihypertensive medication use and 2-year risk of pancreatic cancer diagnosis in the HPFS cohort

	Recent change in medication use [a,b]		
	No change	Start	Stop
Anticoagulant medications			
No. of cases	214	5	4
Person-years	447,083	8,013	3,711
Incidence rate (per 100,000 person-years)	48 (42-55)	62 (26-150)	108 (40-287)
Unadjusted	1 [Ref]	1.30 (0.54-3.16)	2.25 (0.84-6.06)
Age-adjusted [c]	1	1.02 (0.42-2.50)	1.48 (0.53-4.08)
MV-adjusted [d]	1	0.97 (0.39-2.38)	1.22 (0.38-3.90)
MV-adjusted [e] (replace BMI with weight loss)	1	0.94 (0.38-2.31)	1.22 (0.38-3.89)
Antihypertensive medications			
No. of cases	335	28	40
Person-years	860,393	69,599	48,353
Incidence rate (per 100,000 person-years)	39 (35-43)	40 (28-58)	83 (61-113)
Unadjusted	1	1.03 (0.70-1.52)	2.12 (1.53-2.95)
Age-adjusted [c]	1	0.82 (0.55-1.21)	1.50 (1.07-2.11)
MV-adjusted [d]	1	0.79 (0.53-1.18)	1.38 (0.95-2.01)
MV-adjusted [e] (replace BMI with weight loss)	1	0.79 (0.53-1.19)	1.30 (0.89-1.90)

Abbreviations: HPFS, Health Professionals Follow-up Study; MV, multivariate; BMI, body mass index; MET, metabolic equivalent task.

[a] Follow-up time: antidiabetic medications (information on antidiabetic medication use in HPFS was assessed relatively late (in 2008 and afterwards) and was therefore not included in the analyses), anticoagulant (HPFS: 1998-2012), and antihypertensive medications (HPFS: 1988-2012).

[b] Medication change was measured by comparing questionnaires returned at a median of 1 year (current status of use) and 3 years (previous status of use) before pancreatic cancer diagnosis.

[c] Stratified by age (in months) and calendar year of the survey cycle (each 2-year interval).

[d] Stratified by age (in months) and calendar year of the survey cycle (each 2-year interval); adjusted for race/ethnicity (white, black, other, unknown), BMI (continuous, kg/m^2), physical activity (continuous, MET-hours/week), smoking (continuous, pack-years), alcohol intake (continuous, grams/day), history of diabetes (yes, no), multivitamin use (yes, no), and previous status of use (yes, no).

[e] Replace BMI (continuous, kg/m^2) with recent weight loss (no, ≤5%, 5%< to ≤ 10%, and >10% weight loss).

Supplementary Table 11. Recent change in antacid, NSAIDs, and antidepressant medication use and 2-year risk of pancreatic cancer diagnosis in the HPFS cohort

	Recent change in medication use [a,b]		
	No change	Start	Stop
Antacids			
No. of cases	42	5	5
Person-years	94,181	7,227	7,059
Incidence rate (per 100,000 person-years)	45 (33-60)	69 (29-166)	71 (29-170)
Unadjusted	1 [Ref]	1.55 (0.61-3.92)	1.59 (0.63-4.01)
Age-adjusted [c]	1	1.52 (0.59-3.90)	1.44 (0.56-3.70)
MV-adjusted [d]	1	1.29 (0.48-3.47)	1.90 (0.47-7.64)
MV-adjusted [e] (replace BMI with weight loss)	1	1.24 (0.46-3.35)	1.93 (0.48-7.77)
NSAIDs			
No. of cases	268	34	39
Person-years	671,067	91,516	66,548
Incidence rate (per 100,000 person-years)	40 (35-45)	37 (27-52)	59 (43-80)
Unadjusted	1	0.93 (0.65-1.33)	1.47 (1.05-2.05)
Age-adjusted [c]	1	0.99 (0.69-1.42)	1.36 (0.96-1.92)
MV-adjusted [d]	1	0.97 (0.66-1.45)	1.40 (0.98-2.02)
MV-adjusted [e] (replace BMI with weight loss)	1	0.98 (0.66-1.45)	1.39 (0.97-2.00)
Antidepressants			
No. of cases	314	3	9
Person-years	740,060	12,838	8,972
Incidence rate (per 100,000 person-years)	42 (38-47)	23 (8-72)	100 (52-193)
Unadjusted	1	0.55 (0.18-1.72)	2.36 (1.22-4.59)
Age-adjusted [c]	1	0.53 (0.17-1.66)	2.20 (1.12-4.31)
MV-adjusted [d]	1	0.50 (0.16-1.58)	1.02 (0.43-2.42)
MV-adjusted [e] (replace BMI with weight loss)	1	0.49 (0.16-1.55)	0.96 (0.40-2.29)

Abbreviations: HPFS, Health Professionals Follow-up Study; MV, multivariate; BMI, body mass index; MET, metabolic equivalent task; NSAIDs, non-steroidal anti-inflammatory drugs.

[a] Follow-up time: antacids (HPFS: 2006-2012), NSAIDs (HPFS: 1988-2012), and antidepressants (HPFS: 1992-2012).

[b] Medication change was measured by comparing questionnaires returned at a median of 1 year (current status of use) and 3 years (previous status of use) before pancreatic cancer diagnosis.

[c] Stratified by age (in months) and calendar year of the survey cycle (each 2-year interval).

[d] Stratified by age (in months) and calendar year of the survey cycle (each 2-year interval); adjusted for race/ethnicity (white, black, other, unknown), BMI (continuous, kg/m^2), physical activity (continuous, MET-hours/week), smoking (continuous, pack-years), alcohol intake (continuous, grams/day), history of diabetes (yes, no), multivitamin use (yes, no), and previous status of use (yes, no).

[e] Replace BMI (continuous, kg/m^2) with recent weight loss (no, ≤5%, 5%< to ≤ 10%, and >10% weight loss).

Supplementary Table 12. Combined associations of recent changes in medication use with 2-year risk of pancreatic cancer diagnosis in the NHS cohort

	Number of medication changes (starting antidiabetic, starting anticoagulant, and stopping antihypertensive) [a,b,c,d]			*P* for trend [e]
	0	1	≥ 2	
No. of cases	294	76	6	
Person-years	1,025,517	98,969	3,668	
Incidence rate (per 100,000 person-years)	29 (26-32)	77 (61-96)	164 (73-364)	
Unadjusted	1 [Ref]	2.68 (2.08-3.45)	5.71 (2.54-12.8)	$<1\times10^{-5}$
Age-adjusted [f]	1	2.30 (1.78-2.98)	4.04 (1.78-9.17)	$<1\times10^{-5}$
MV-adjusted [g]	1	1.89 (1.41-2.52)	3.05 (1.24-7.50)	$<1\times10^{-5}$
MV-adjusted [h] (replace BMI with weight loss)	1	1.76 (1.32-2.36)	2.72 (1.09-6.77)	5×10^{-5}

Abbreviations: NHS, Nurses' Health Study; MV, multivariate; BMI: Body Mass Index; MET, metabolic equivalent task.

[a] Information on antidiabetic medication use in HPFS was assessed relatively late (in 2008 and afterwards), and therefore HPFS data was not included in the analyses of combined associations of starting antidiabetic medications, staring anticoagulant, and stopping antihypertensive medications.

[b] Follow-up time: combined associations of starting antidiabetic medications, staring anticoagulant, and stopping antihypertensive. Baseline was defined as 1996 because 1994 was the first year when information on use of antidiabetic medications, anticoagulant, and antihypertensive medications were all available.

[c] Medication change was measured by comparing questionnaires returned at a median of 1 year (current status of use) and 3 years (previous status of use) before pancreatic cancer diagnosis.

[d] Medication changes considered in this analysis include the start of antidiabetic or anticoagulant medications and the stop of antihypertensive medications.

[e] Two-sided *P* value for trend was calculated by entering the number of medication changes as an ordinal variable and assessed by the Wald test without correction for multiple comparisons. *P* value $<1\times10^{-5}$ for trend in MV-adjusted analysis.

[f] Stratified by age (in months) and calendar year of the survey cycle (each 2-year interval).

[g] Stratified by age (in months) and calendar year of the survey cycle (each 2-year interval); adjusted for race/ethnicity (white, black, other, unknown), BMI (continuous, kg/m^2), physical activity (continuous, MET-hours/week), smoking (continuous, pack-years), alcohol intake (continuous, grams/day), history of diabetes (yes, no), multivitamin use (yes, no), and previous status of use (yes, no).

[h] Replace BMI (continuous, kg/m^2) with recent weight loss (no, ≤5%, 5%< to ≤ 10%, and >10% weight loss).

Supplementary Table 13. Recent change in medication use and 2-year risk of other digestive system cancer diagnoses in the pooled NHS and HPFS cohorts [a]

	Recent change in medication use [b,c]		
	No change	Start	Stop
Antidiabetic medications			
No. of cases	2,524	65	35
Person-years	1,953,339	33,133	15,474
Incidence rate (per 100,000 person-years)	129 (124-134)	196 (154-250)	226 (162-315)
Unadjusted	1 [Ref]	1.52 (1.19-1.94)	1.75 (1.25-2.44)
Age-adjusted [d]	1	1.33 (1.04-1.70)	1.33 (0.95-1.87)
MV-adjusted [e]	1	1.09 (0.83-1.44)	1.04 (0.70-1.54)
MV-adjusted [f] (replace BMI with weight loss)	1	1.11 (0.84-1.47)	1.01 (0.68-1.50)
Oral hypoglycemic medications			
No. of cases	2538	48	38
Person-years	1,961,622	26,498	13,825
Incidence rate (per 100,000 person-years)	129 (124-135)	181 (137-240)	275 (200-378)
Unadjusted	1	1.40 (1.05-1.86)	2.12 (1.54-2.93)
Age-adjusted [d]	1	1.23 (0.92-1.64)	1.63 (1.17-2.26)
MV-adjusted [e]	1	1.00 (0.73-1.37)	1.36 (0.91-2.01)
MV-adjusted [f] (replace BMI with weight loss)	1	1.02 (0.74-1.39)	1.33 (0.90-1.97)
Insulin			
No. of cases	2598	21	5
Person-years	1,989,111	8,593	4,241
Incidence rate (per 100,000 person-years)	131 (126-136)	244 (159-375)	118 (49-283)
Unadjusted	1	1.87 (1.22-2.87)	0.90 (0.38-2.17)
Age-adjusted [d]	1	1.63 (1.06-2.51)	0.71 (0.29-1.71)
MV-adjusted [e]	1	1.31 (0.84-2.04)	0.51 (0.20-1.30)
MV-adjusted [f] (replace BMI with weight loss)	1	1.35 (0.86-2.10)	0.50 (0.20-1.26)
Anticoagulant medications			
No. of cases	2741	55	22
Person-years	1,743,068	25,141	12,050
Incidence rate (per 100,000 person-years)	157 (151-163)	219 (168-285)	183 (120-277)
Unadjusted	1	1.39 (1.07-1.82)	1.16 (0.76-1.77)
Age-adjusted [d]	1	1.09 (0.83-1.42)	0.88 (0.58-1.35)
MV-adjusted [e]	1	1.05 (0.80-1.37)	0.63 (0.40-1.01)
MV-adjusted [f] (replace BMI with weight loss)	1	1.05 (0.80-1.37)	0.63 (0.40-1.00)
Antihypertensive medications			
No. of cases	3587	371	269
Person-years	2,610,059	222,169	140,215
Incidence rate (per 100,000 person-years)	137 (133-142)	167 (151-185)	192 (170-216)
Unadjusted	1	1.22 (1.09-1.35)	1.40 (1.23-1.58)
Age-adjusted [d]	1	1.07 (0.96-1.19)	1.09 (0.96-1.24)
MV-adjusted [e]	1	1.05 (0.94-1.17)	1.04 (0.91-1.19)
MV-adjusted [f] (replace BMI with weight loss)	1	1.08 (0.96-1.20)	1.02 (0.89-1.17)

Abbreviations: NHS, Nurses' Health Study; HPFS, Health Professionals Follow-up Study; MV, multivariate; BMI, body mass index; MET, metabolic equivalent task.

[a] Other cancers of the digestive system, including cancers of esophagus, stomach, small intestine, colorectum, anus, liver, and gallbladder.

[b] Follow-up time: antidiabetic medications (NHS: 1990-2012; information on antidiabetic medication use in HPFS was assessed relatively late (in 2008 and afterwards) and was therefore not included in the analyses), anticoagulant (NHS: 1996-2012; HPFS: 1998-2012), and antihypertensive medications (NHS: 1990-2012; HPFS: 1988-2012).

[c] Medication change was measured by comparing questionnaires returned at a median of 1 year (current status of use) and 3 years (previous status of use) before pancreatic cancer diagnosis.

[d] Stratified by age (in months), sex/cohort (women, men; in the pooled analyses only), and calendar year of the survey cycle (each 2-year interval).

[e] Stratified by age (in months), sex/cohort (women, men; in the pooled analyses only), and calendar year of the survey cycle (each 2-year interval); adjusted for race/ethnicity (white, black, other, unknown), BMI (continuous, kg/m^2), physical activity (continuous, MET-hours/week), smoking (continuous, pack-years), alcohol intake (continuous, grams/day), history of diabetes (yes, no), multivitamin use (yes, no), and previous status of use (yes, no).

[f] Replace BMI (continuous, kg/m^2) with recent weight loss (no, ≤5%, 5%< to ≤ 10%, and >10% weight loss).
