# Supplementary material for: Pancreatic cancer is associated with medication changes prior to clinical diagnosis
Source: Nat Commun. 2023 Apr 28;14:2437. doi: 10.1038/s41467-023-38088-2 (PMC10147931; doi:10.1038/s41467-023-38088-2)
Supplement: Supplementary file 3 — Reporting Summary [file 41467_2023_38088_MOESM3_ESM.pdf]

nature portfolio

Corresponding author(s): Brian M. Wolpin

Last updated by author(s): Mar 21, 2023

Reporting Summary

Nature Portfolio wishes to improve the reproducibility of the work that we publish. This form provides structure for consistency and transparency in reporting. For further information on Nature Portfolio policies, see our Editorial Policies and the Editorial Policy Checklist.

Statistics

For all statistical analyses, confirm that the following items are present in the figure legend, table legend, main text, or Methods section.

n/a	Confirmed	
☐	☒	The exact sample size (n) for each experimental group/condition, given as a discrete number and unit of measurement
☐	☒	A statement on whether measurements were taken from distinct samples or whether the same sample was measured repeatedly
☐	☒	The statistical test(s) used AND whether they are one- or two-sided<br>*Only common tests should be described solely by name; describe more complex techniques in the Methods section.*
☐	☒	A description of all covariates tested
☐	☒	A description of any assumptions or corrections, such as tests of normality and adjustment for multiple comparisons
☐	☒	A full description of the statistical parameters including central tendency (e.g. means) or other basic estimates (e.g. regression coefficient) AND variation (e.g. standard deviation) or associated estimates of uncertainty (e.g. confidence intervals)
☐	☒	For null hypothesis testing, the test statistic (e.g. F, t, r) with confidence intervals, effect sizes, degrees of freedom and P value noted<br>*Give P values as exact values whenever suitable.*
☒	☐	For Bayesian analysis, information on the choice of priors and Markov chain Monte Carlo settings
☐	☒	For hierarchical and complex designs, identification of the appropriate level for tests and full reporting of outcomes
☒	☐	Estimates of effect sizes (e.g. Cohen's d, Pearson's r), indicating how they were calculated

Our web collection on statistics for biologists contains articles on many of the points above.

Software and code

Policy information about availability of computer code

Data collection	No software was used for data collection.
Data analysis	Data analyses were performed using SAS statistical software, version 9.4 for UNIX (SAS Institute Inc., Cary, NC). The analysis programs are publicly available through https://github.com/drzhangyin/med_change_pdac.

For manuscripts utilizing custom algorithms or software that are central to the research but not yet described in published literature, software must be made available to editors and reviewers. We strongly encourage code deposition in a community repository (e.g. GitHub). See the Nature Portfolio guidelines for submitting code & software for further information.

Data

Policy information about availability of data

All manuscripts must include a data availability statement. This statement should provide the following information, where applicable:

- Accession codes, unique identifiers, or web links for publicly available datasets
- A description of any restrictions on data availability
- For clinical datasets or third party data, please ensure that the statement adheres to our policy

Data described in the manuscript are available upon formal application to and approval by the Channing Division of Network Medicine at Brigham and Women's Hospital, and Harvard T.H. Chan School of Public Health. To ensure the confidentiality and privacy of cohort participants, written request for access to the data is required. The standard procedure for controlled access requires that applications to use the resources of the Nurses' Health Studies and Health Professionals Follow-up Study undergo a formal review by the cohort committee. The committee assesses the scientific aims, examines the suitability of the proposed

methodology for the available data, and confirms that the proposed use aligns with the guidelines of the Ethics and Governance Framework. Further information including the procedures to obtain and access data from the Nurses' Health Study and Health Professionals Follow-Up Study is described at https://www.nurseshealthstudy.org/researchers (email: nhsaccess@channing.harvard.edu) and https://sites.sph.harvard.edu/hpfs/for-collaborators/.

Human research participants

Policy information about studies involving human research participants and Sex and Gender in Research.

Reporting on sex and gender	Our study follows the 'Sex and Gender Equity in Research – SAGER – guidelines' and includes sex and gender considerations where relevant.
Population characteristics	The study population included participants from two large U.S. longitudinal cohorts: the Nurses Health Study (NHS) and Health Professionals Follow-up Study (HPFS). The NHS began in 1976, enrolling 121,700 US female nurses between 30 and 55 years of age. HPFS was initiated in 1986,21 when 51,529 US male health professionals aged 40 to 75 years were included. Participants with prior history of cancer were excluded at baseline. We also excluded survey cycles with missing medication information reported by participants. We included up to 148,973 eligible participants in final analyses.
Recruitment	The NHS began in 1976, enrolling 121,700 US female nurses between 30 and 55 years of age. HPFS was initiated in 1986,21 when 51,529 US male health professionals aged 40 to 75 years were included.
Ethics oversight	The protocols were approved by the Institutional Review Boards of the Brigham and Women's Hospital (Boston, MA) and the Harvard T.H. Chan School of Public Health (Boston, MA), and those of participating registries as required. Written informed consent was required to retrieve medical records.

Note that full information on the approval of the study protocol must also be provided in the manuscript.

Field-specific reporting

Please select the one below that is the best fit for your research. If you are not sure, read the appropriate sections before making your selection.

☐ Life sciences ☒ Behavioural & social sciences ☐ Ecological, evolutionary & environmental sciences

For a reference copy of the document with all sections, see nature.com/documents/nr-reporting-summary-flat.pdf

Behavioural & social sciences study design

All studies must disclose on these points even when the disclosure is negative.

Study description	Quantitative prospective cohort study
Research sample	The study population included participants from the NHS and HPFS. Briefly, the NHS began in 1976, enrolling 121,700 US female nurses between 30 and 55 years of age. The HPFS was initiated in 1986,21 when 51,529 US male health professionals aged 40 to 75 years were included. The study sample is representative of U.S. health professionals and includes both sexes. Healthcare professionals have the health knowledge and ability to provide complete and accurate information on questionnaires, and they are more likely to maintain participation in a long-term study (decades of follow-up) than the general U.S. population. This study sample has several other major strengths of note. For example, it allows simultaneously examining several types of medications in two prospective studies with up to 148,973 eligible participants and 24 years of follow-up. In addition, the prospective study design with enrollment prior to cancer diagnosis in conjunction with very low rates of loss to follow-up minimize the potential for exposure misclassification, recall bias and issues with patient selection. Moreover, validated time-varying information on a wide spectrum of covariates permits rigorous control for known or plausible confounding.
Sampling strategy	The NHS participants are female, married registered nurse (birth date between January 1, 1921 and December 31, 1946) residing in the 11 states with the largest number of registered nurses (New York, California, Pennsylvania, Ohio, Massachusetts, New Jersey, Michigan, Texas, Florida, Connecticut, and Maryland). The HPFS participants are male veterinarians dentists, pharmacists, optometrists, osteopath physicians, & podiatrists between the ages of 40-75 at baseline. At cohort enrollment in the NHS in 1976, there were three mailings of the baseline questionnaire (first mailing to all 238,026 nurses, followed by second and third mailings to non-respondents), and the final cohort number of participants was established after cleaning the original data of duplicates and ineligible respondents. Similar sampling approach was used in the HPFS. The study population is preexisting. We investigated this research topic in both cohorts, including up to 148,973 eligible participants followed for 2,994,057 person-years and up to 991 incident pancreatic cancer cases. This very large sample size is sufficient to detect the major findings of the present study.
Data collection	In both cohorts, participant demographics were assessed from returned questionnaires, including age, sex, and race/ethnicity. Participants biennially updated medication use, weight, cigarette smoking, physical activity, history of diabetes mellitus, and history of multivitamin use. Dietary data, including information on alcohol intake, were updated quadrennially via validated, semi-quantitative food frequency questionnaires. Cohort staff collecting and managing the data were blinded to the hypothesis.
Timing	In both cohorts, each questionnaire is mailed over a 2-year questionnaire cycle. The first mailing of the new questionnaire is in June (even years).

	Timelines of exposure assessments:<br>Antidiabetic medications (NHS: 1988-2010)<br>Anticoagulant medications (NHS: 1994-2010; HPFS: 1996-2010)<br>Antihypertensive medications (NHS: 1988-2010; HPFS: 1986-2010)<br>Antacids (NHS: 2000-2010; HPFS: 2004-2010)<br>NSAIDs (NHS: 1990-2010; HPFS: 1986-2010)<br>Antidepressants (NHS: 1996-2010; HPFS: 1990-2010)<br>Combined antidiabetic, anticoagulant, and antihypertensive medications (NHS: 1994-2010)<br><br>Timelines of analyses :<br>Antidiabetic medications (NHS: 1990-2012)<br>Anticoagulant medications (NHS: 1996-2012; HPFS: 1998-2012)<br>Antihypertensive medications (NHS: 1990-2012; HPFS: 1988-2012)<br>Antacids (NHS: 2002-2012; HPFS: 2006-2012)<br>NSAIDs (NHS: 1992-2012; HPFS: 1988-2012)<br>Antidepressants (NHS: 1998-2012; HPFS: 1992-2012)<br>Combined antidiabetic, anticoagulant, and antihypertensive medications (NHS: 1996-2012)
Data exclusions	Participants with prior history of cancer were excluded at baseline. We also excluded survey cycles with missing medication information reported by participants. We included up to 148,973 eligible participants in final analyses.
Non-participation	The NHS and HPFS cohorts have achieved a high follow-up rate exceeding 90% throughout decades of follow-up.
Randomization	Not applicable. This study is an observational prospective cohort study. There are no experimental groups or interventions.

Reporting for specific materials, systems and methods

We require information from authors about some types of materials, experimental systems and methods used in many studies. Here, indicate whether each material, system or method listed is relevant to your study. If you are not sure if a list item applies to your research, read the appropriate section before selecting a response.

Materials & experimental systems

n/a	Involved in the study
☒	☐ Antibodies
☒	☐ Eukaryotic cell lines
☒	☐ Palaeontology and archaeology
☒	☐ Animals and other organisms
☒	☐ Clinical data
☒	☐ Dual use research of concern

Methods

n/a	Involved in the study
☒	☐ ChIP-seq
☒	☐ Flow cytometry
☒	☐ MRI-based neuroimaging
